# Centers of endemism of freshwater protists deviate from pattern of taxon richness on a continental scale

**DOI:** 10.1038/s41598-020-71332-z

**Published:** 2020-09-02

**Authors:** Jana L. Olefeld, Christina Bock, Manfred Jensen, Janina C. Vogt, Guido Sieber, Dirk Albach, Jens Boenigk

**Affiliations:** 1 Biodiversity, University of Duisburg-Essen, Universitätsstr. 5, 45141 Essen, Germany; 2 Institute for Biology and Environmental Science (IBU), Plants Biodiversity and Evolution, Carl Von Ossietzky University, Carl-von-Ossietzky-Str. 9-11, 26129 Oldenburg, Germany

**Keywords:** Biogeography, Freshwater ecology, Microbial ecology, Water microbiology, Biodiversity

## Abstract

Here, we analyzed patterns of taxon richness and endemism of freshwater protists in Europe. Even though the significance of physicochemical parameters but also of geographic constraints for protist distribution is documented, it remains unclear where regional areas of high protist diversity are located and whether areas of high taxon richness harbor a high proportion of endemics. Further, patterns may be universal for protists or deviate between taxonomic groups. Based on amplicon sequencing campaigns targeting the SSU and ITS region of the rDNA we address these patterns at two different levels of phylogenetic resolution. Our analyses demonstrate that protists have restricted geographical distribution areas. For many taxonomic groups the regions of high taxon richness deviate from those having a high proportion of putative endemics. In particular, the diversity of high mountain lakes as azonal habitats deviated from surrounding lowlands, i.e. many taxa were found exclusively in high mountain lakes and several putatively endemic taxa occurred in mountain regions like the Alps, the Pyrenees or the Massif Central. Beyond that, taxonomic groups showed a pronounced accumulation of putative endemics in distinct regions, e.g. Dinophyceae along the Baltic Sea coastline, and Chrysophyceae in Scandinavia. Many other groups did not have pronounced areas of increased endemism but geographically restricted taxa were found across Europe.

## Introduction

Restricted distribution and endemism has been demonstrated for numerous protist taxa by now^1–4^. However, the geographic patterns underlying protist distribution are not yet resolved. Here, we aim at disentangling areas of high and low protist diversity on a European scale and further test dependencies between local taxon richness and local accumulation of endemics.

Many factors influence the diversity and distribution of species, resulting in a heterogeneously distributed taxon richness across the world^5^. For microscopic species the assumption of a ubiquitous distribution changed towards a general acceptance of a “moderate endemicity”^6–8^. Even though restricted dispersal and distribution pattern at least for some taxa are unquestioned^9–12^ and large-scale variation between protist communities have been shown^13–15^ the resulting pattern as well as areas of high and low protist diversity are largely unresolved. Distinct habitats may differ considerably in taxon richness^14,16–18^, which is often related to environmental factors such as pH, altitude and trophy. Examples for regional differences in taxon richness of animals and plants as well as of protists are mountain ranges^18–22^ which, however, basically reflect effects of one of the formerly mentioned abiotic factors, i.e. altitude and covarying factors, rather than geographic differentiation. Even though high mountain ranges have been suggested to hinder dispersal and thus to structure protist diversity between remote regions^7,23^, taxon richness on both sides of mountain ranges do not necessarily differ despite the above-mentioned altitudinal gradients within the mountain ranges^18^. Aside from such azonal areas and special habitats, it remains unclear where regional areas of high protist diversity are located and whether these correspond to regions of high biodiversity known from macro-organisms. It is further unclear whether such areas are universally valid for all taxonomic groups or specific for distinct taxa. Only for a few taxonomic groups a regionally outstandingly high species richness has so far been suggested and only for few regions such as the Aquitaine region (France) for Synurales (Chrysophyceae)^24^ or the Antarctic and sub-Antarctic for *Stauroneis* (Bacillariophyta) floras^25^. The importance both of geographic distance and of geographic barriers for structuring pattern of protist taxon richness and in particular the relative importance of these two factors on continental scales remain disputable.

Likewise, it is unclear where regional centers of protist endemism are located and whether these regions correspond to areas of high protist taxon richness. Mechanisms allowing for a high local or regional taxon richness can be different from those which restrict dispersal^26,27^. For instance, local habitat heterogeneity allows for a high regional taxon diversity^28^ whereas geographic barriers are considered to limit dispersal^29^. Areas with high numbers of endemic protist taxa, thus, can but do not necessarily merely covary with taxon richness, i.e. regions of high taxon richness may deviate from regions holding high numbers of endemic taxa as it has been inferred for animals and plants^30,31^. For protists such relations between taxon richness and endemism are not known. To date it is unclear (i) where to expect regions of high protist taxon richness and (ii) where to expect regions holding high numbers of endemic protist taxa. Studies so far strongly center around azonal habitats and regions with island character: In particular, high mountain ranges have been suggested to act as islands for endemic taxa both for macro- and microorganisms^32,33^. Further, elevation seems to be a crucial factor structuring species distribution and diversity for animals and plants, but also for protists^18,20,22,34^.

Aside from such special and azonal habitats, investigations for centers of protist endemism are again scarce and mostly restricted to local or regional studies on selected taxonomic groups^24,25^. In the marine environment (e.g.^15,16,35^) and to a lesser extent also in fresh waters (e.g.^14,18,27,36^) recent large scale surveys revealed some trends of protist distribution but general patterns of protist endemism in fresh waters are still unclear on continental scales. It is even more vague whether areas of high endemism are congruent for different taxonomic groups. Here, we thus analyze congruencies and discrepancies between patterns of taxon richness and endemism for protists and differences between taxonomic groups.

Signs of restricted distribution may already be apparent for subpopulations below species level, i.e. distribution areas of the subpopulations may be considerably smaller than that of the taxon^13,37,38^. Consequently, beyond the analysis of SSU-based OTU diversity we further applied paired sequencing of SSU and ITS sequences to test whether geographic constraints also apply to populations unresolved by SSU and thus address the issue of population structure and population genetics of free-living protists. In contrast to the relatively conserved SSU rRNA, the ITS-region is highly variable and allows differentiation of very closely related species or of intraspecific subpopulations^39,40^. Unfortunately, large differences in sequence length and the high degree of sequence diversity complicates analyses of these region^7,41^. A dataset with population-like structure can be generated using the SSU-V9- and ITS1-region, resulting in a microdiversity of ITS-sequences with identical V9-sequences differing in their geographic distribution^13^.

We here hypothesize that (i) areas of high taxon diversity can be separated from areas of low taxon diversity, (ii) taxa (as well as subpopulations of taxa) tend to have a restricted geographic distribution and are not randomly distributed across Europe, (iii) patterns of protist endemism deviate from patterns of protist taxon richness and that (iv) these patterns vary between taxonomic groups. We further hypothesize that geographic distance and geographic barriers differentially affect distribution pattern.

## Results

Filtering resulted in a dataset comprising 13,640 V9-groups with 285,755 ITS-variants occurring in 217 natural freshwater lakes. After clustering of the ITS-variants 46,302 ITS-SWARMS remained for analyses.

### Taxa based on V9-sequences (V9-groups) are geographically restrained

**Figure 1:**
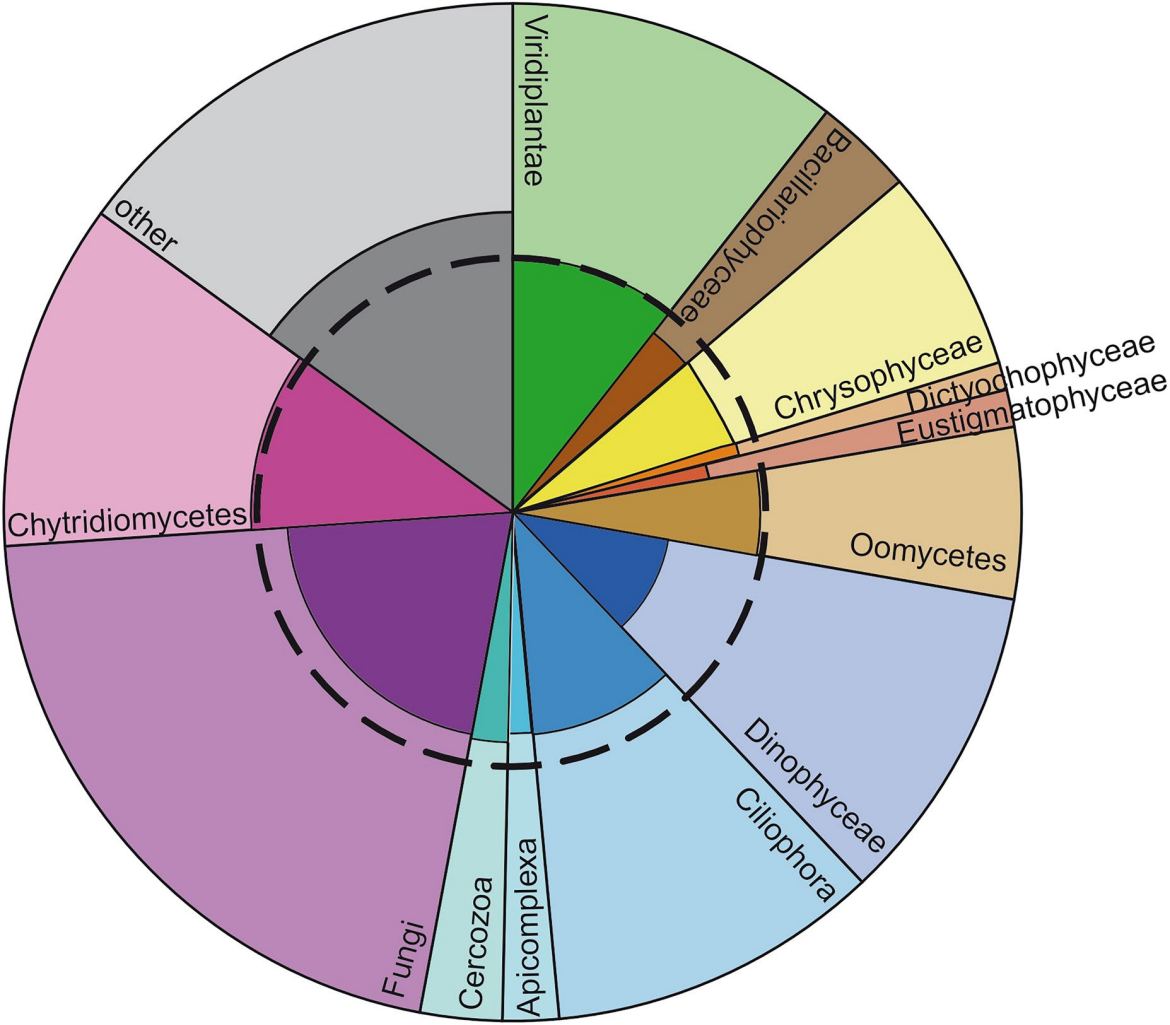
Relative read abundance of different taxonomic groups and fraction of V9-groups showing a geographically restricted occurrence (dark shaded) for each of these groups. The dashed line indicates the 50% line. Graphic was created using R with the package ggplot2 (https://cran.r-project.org/web/packages/ggplot2/index.html) and manually edited with Adobe Illustrator2020.

56% (7,643) of V9-groups were only found in a single lake, in particular Dinophyceae had a noticeably high number of V9-groups, which occurred in only one lake. The majority of OTUs, which occurred in more than one lake, showed indications for a geographically restricted distribution (distribution maps of V9-groups, which occurred in at least 5 lakes, are available under https://doi.org/10.5281/zenodo.3674717), i.e. the mean distance between the respective lakes was smaller than would have been expected for random distribution (p < 0.001; Fig. 1, Table 1). Hence, a geographical restriction of protist V9-groups is present in many taxa, but the extent of geographical restriction varies between taxonomic groups.

**Table 1:** Diversity of V9 groups and ITS-SWARMS as reflected by the richness and the number of taxa with restricted or even putatively endemic distribution. The number of V9 groups comprising ecologically differently adapted subpopulation as reflected by ITS-SWARMS are provided with respect to pH, conductivity, temperature and altitude. As this latter analysis was restricted to V9 groups only which occurred in several lakes and comprised several ITS-SWARMs, the total number of V9 groups subjected to this analysis is also given for comparison.

Taxonomy	Biogeographic analyses					
	V9-groups (total)	ITS-SWARMS (total)	Average ITS-SWARMS/V9-group	Geographical restricted V9-groups	Geographical restricted ITS-SWARMS	Putativly endemic V9-groups
Chloroplastida	635 (1,080)	1666 (4,432)	2.62 (4.10)	315 (50%)	859 (52%)	65 (10%)
Bacillariophyceae	189 (394)	902 (3,199)	4.78 (8.12)	81 (43%)	552 (61%)	21 (11%)
Chrysophyceae	390 (775)	1,260 (4,741)	3.23 (6.12)	172 (44%)	710 (56%)	42 (11%)
Dictyochophyceae	53 (80)	455 (1,245)	8.58 (15.56)	24 (45%)	302 (66%)	6 (11%)
Eustigmatophyceae	71 (170)	241 (844)	3.39 (4.96)	28 (39%)	144 (60%)	2 (3%)
Oomycetes	326 (597)	557 (1522)	1.71 (2.55)	158 (48%)	262 (47%)	30 (9%)
Dinophyceae	612 (1755)	1,415 (4,878)	2.31 (2.78)	186 (30%)	588 (42%)	50 (8%)
Ciliophora	635 (1,219)	1,395 (3,641)	2.20 (2.20)	286 (45%)	630 (45%)	62 (10%)
Apicomplexa	107 (280)	273 (1,071)	2.55 (3.83)	46 (43%)	130 (48%)	11 (10%)
Cercozoa	156 (418)	291 (1,226)	1.87 (2.93)	71 (46%)	142 (49%)	27 (17%)
Fungi	1,260 (3,044)	2026 (7,918)	1.61 (2.60)	556 (44%)	867 (43%)	140 (11%)
Chytridiomycetes	664 (1537)	1,245 (4,109)	1.88 (2.67)	347 (52%)	535 (43%)	88 (13%)

Taxonomy	Environmental analyses					
	V9-groups comprising differently adapted ITS-SWARMS					
	Examined V9-groups	pH	Conductivity	Temperature	Altitude	
Chloroplastida	419	7 (1.67%)	19 (4.53%)	13 (3.10%)	9 (2.15%)	
Bacillariophyceae	155	2 (1.29%)	6 (3.87%)	6 (3.87%)	7 (4.52%)	
Chrysophyceae	299	5 (1.67%)	13 (4.34%)	12 (4.01%)	7 (2.34%)	
Dictyochophyceae	40	3 (7.50%)	3 (7.50%)	5 (12.50%)	3 (7.50%)	
Eustigmatophyceae	41	0 (0 .00%)	0 (0.00%)	1 (2.44%)	1 (2.44%)	
Oomycetes	226	3 (1.33%)	2 (0.88%)	2 (0.88%)	4 (1.77%)	
Dinophyceae	288	6 (2.08%)	9 (3.13%)	9 (3.13%)	8 (2.78%)	
Ciliophora	384	9 (2.34%)	9 (2.34%)	11 (2.86%)	10 (2.60%)	
Apicomplexa	69	1 (1.45%)	2 (3.00%)	2 (3.00%)	1 (1.45%)	
Cercozoa	103	1 (0.97%)	3 (2.91%)	2 (1.94%)	1 (0.97%)	
Fungi	824	4 (0.49%)	5 (0.61%)	4 (0.49%)	10 (1.21%)	
Chytridiomycetes	394	3 (0.76%)	5 (1.27%)	9 (2.28%)	16 (4.06%)	

For instance, 52% of the V9-groups affiliated with Chytridiomycetes and 44% of the V9-groups affiliated with Fungi were geographical restricted. In contrast, only 30% of the V9-groups affiliated with Dinophyceae were geographically restricted. For all other taxa the fraction of geographically restricted V9-groups ranged between 39% (Eustigmatophyceae) and 50% (Chloroplastida) (Table 1).

### Regions of high taxon richness (V9-richness) differ between taxonomic groups

**Figure 2:**
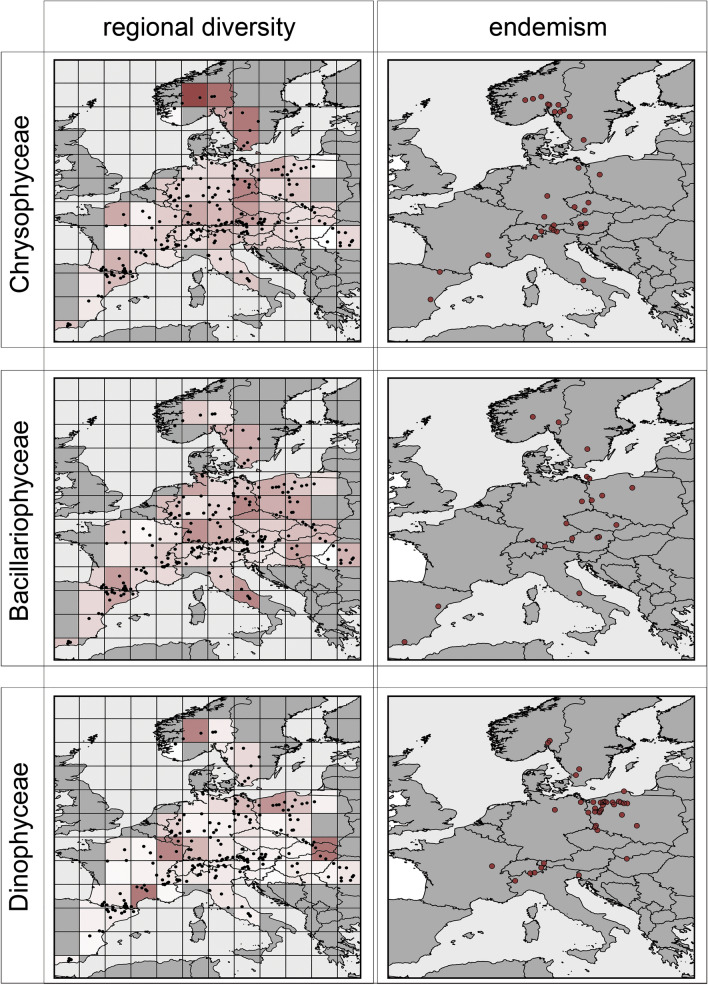
Regional diversity of Chrysophyceae, Bacillariophyceae and Dinophyceae. Left: Regional richness of the respective taxon where color shading indicates low (white) to high (red) regional richness. Maximal shading corresponds to 23 interpolated V9-groups per lake for Chrysophyceae, to 15.4 interpolated V9-groups per lake for Bacillariophyceae and to 17.5 interpolated V9-groups per lake for Dinophyceae. Right: centers of distribution areas for putatively endemic taxa within the respective group. Please note that the centers of the distribution area are geometric centers and do not coincide with a location of a distinct lake. Maps were created using *R* with package *rworldmap* v. 1.3–6 (https://cran.r-project.org/web/packages/rworldmap/index.html) and modified using ggplot2 (https://cran.r-project.org/web/packages/ggplot2/index.html) and Adobe Illustrator2020.

**Figure 3:**
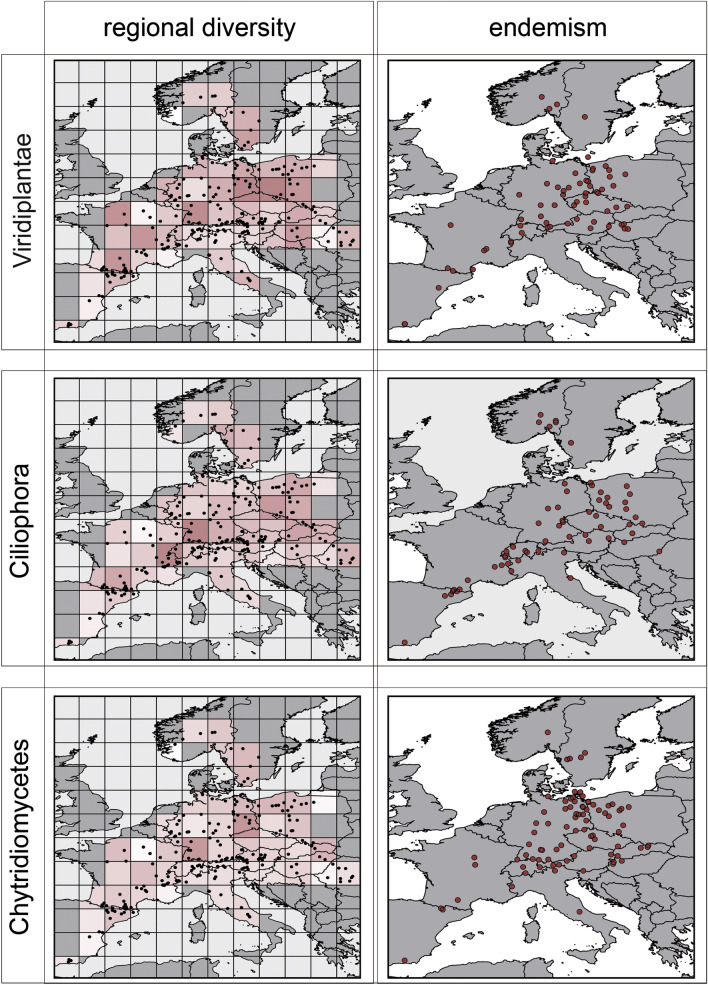
Regional diversity of Viridiplantae (green algae), Ciliophora and Chytridiomycetes. Left: Regional richness of the respective taxon where color shading indicates low (white) to high (red) regional richness. Maximal shading corresponds to 15.4 interpolated V9-groups per lake for Viridiplantae, to 15.1 interpolated V9-groups per lake for Ciliophora and to 13.3 interpolated V9-groups per lake for Chytridiomycota. Right: centers of distribution areas for putatively endemic taxa within the respective group. Please note that the centers of the distribution area are geometric centers and do not coincide with a location of a distinct lake. Maps were created using *R* with package *rworldmap* v. 1.3–6 (https://cran.r-project.org/web/packages/rworldmap/index.html) and modified using ggplot2 (https://cran.r-project.org/web/packages/ggplot2/index.html) and Adobe Illustrator2020.

Diversity patterns vary between protist groups both with respect to taxonomy and with respect to the nutritional strategy. Differential patterns of taxon richness are particularly pronounced for Dinophyceae and Chrysophyceae (Fig. 2). Whereas Dinophyceae do have regions of high taxon richness located around the Massif Central and towards Eastern Europe, Chrysophyceae have a high taxon richness in Scandinavia (Fig. 2). Less pronounced but still a significant unequal distribution of taxon richness was found for phototrophic and mixotrophic taxa, in particular for Viridiplantae and Bacillariophyceae (Figs. 2, 3), Dictyochophyceae and Eustigmatophyceae (Figure S2). Taxon richness was more homogeneously distributed across Europe for heterotrophic and substrate-bound taxa, such as Ciliophora, Apicomplexa, Fungi, Cercozoa and Chytridiomycetes (Figs. 2, 3, S1). An exception were the Oomycetes with a more unevenly distributed pattern of taxon richness. Interestingly, all taxa that do show a more homogenous distribution of taxon richness had a pronounced peak of taxon richness in the grid including Lake Constance.

### Centers of (putative) endemism (based on V9-groups) deviate from regions of high taxon richness

The number of V9-groups with putatively endemic distribution (see methods for definition in the context of this study) varies greatly between the groups studied. Only a very small proportion of the analyzable V9-groups with putative endemic distribution was found within the Eustigmatophyceae (3%), whereas nearly one quarter (17%) of the V9-groups affiliated with Cercozoa met our criteria for putatively endemic distribution (Table 1).

Similar to patterns of taxon richness, regions harboring a particularly high number of groups with putatively endemic distribution differed between taxonomic groups and nutritional strategies but did not necessarily overlap with observed richness patterns (for correlation see Figure S3). In general, many taxa with restricted distribution occurred in mountain regions like the Alps, the Pyrenees or the Massif Central (Figs. 2 and 3 and Figures S1, S2, S8). Beyond such general areas with many putatively endemic taxa, some taxonomic groups were pronouncedly accumulated in distinct regions: For Dinophyceae a region with a high number of putatively endemic taxa is the area near the Baltic Sea coastline (Fig. 2). This clearly deviates from their richness pattern, since taxon richness in this area is only slightly higher compared to other regions. Chrysophyceae exhibited a very different pattern with both, a high number of putatively endemic taxa and a high regional taxon richness in Scandinavia (Fig. 2). The Viridiplantae, Fungi, Oomycetes, Chytridiomycetes and Cercozoa did not show specific areas with taxa of restricted distribution but these were found across the whole sampling area (Fig. 3 and Figures S1 and S2). This was, in principle, similar in diatoms but in this taxon the fraction of taxa with putatively endemic distribution was remarkably low in general. For Ciliates, centers of taxa with putatively endemic distribution are condensed along the Pyrenees and Alps but start to fan out towards Eastern Europe (Fig. 3). For Dictyochophyceae and Eustigmatophyceae only a small number of endemic taxa with putatively restricted distribution were found (Figure S2).

### High mountain ranges are home to many specialist taxa

**Figure 4:**
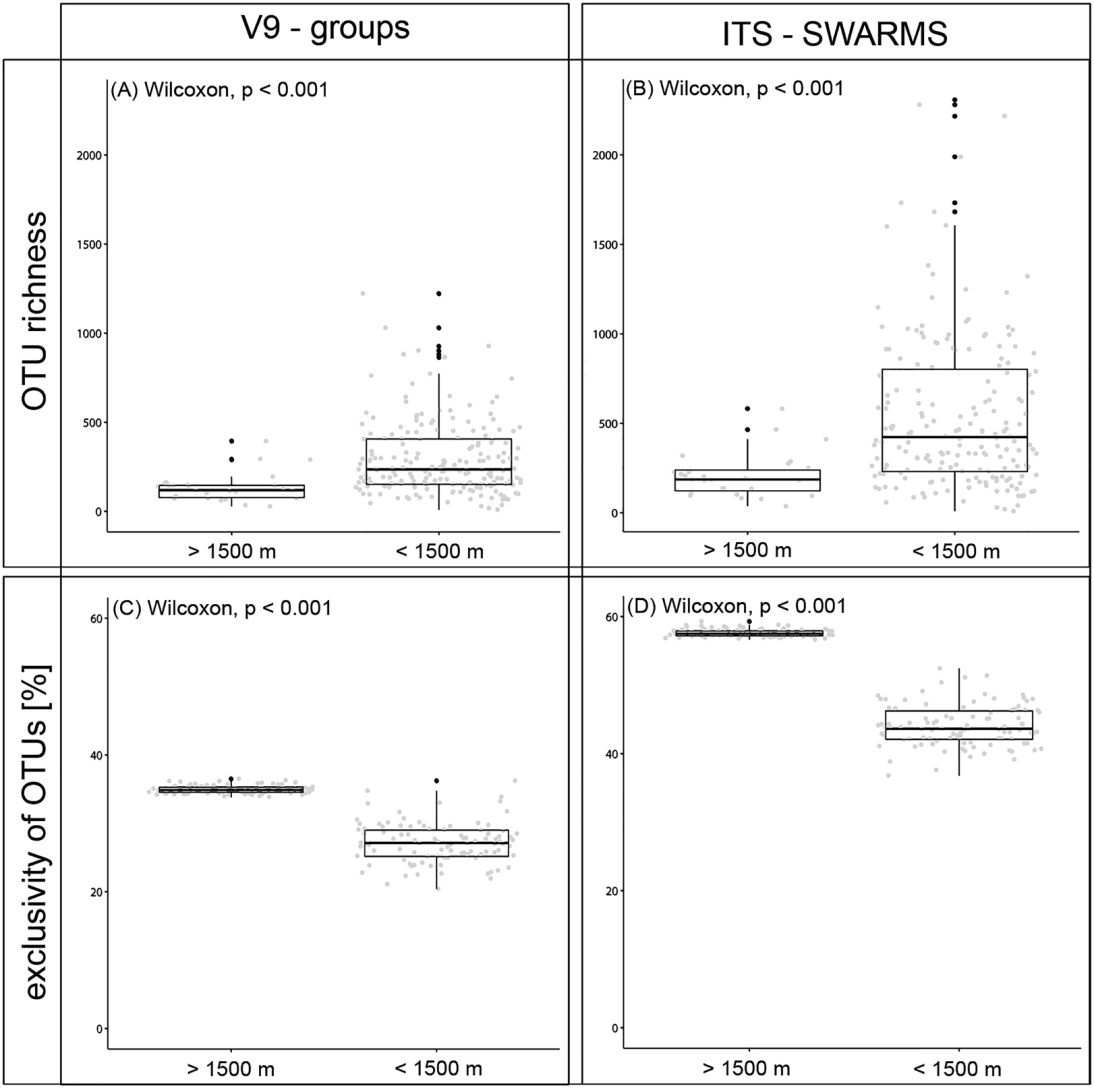
OTU richness (upper graphs) and exclusivity of OTUs (lower graphs) to either high altitude (above 1,500 m) or low altitude (below 1,500 m) lakes. Richness and exclusivity are shown for two different phylogenetic resolutions, i.e. for V9 groups (left) and for ITS-SWARMS (right). Richness is lower but exclusivity is higher in lakes above 1,500 m both for V9-groups and for ITS-SWARMS.

Taxon richness differed strongly between mountain ranges (altitude > 1,500 m) and lowland (altitude < 1,500 m) regions. Significantly lower richness was found in mountain ranges (V9-groups: p = 9.7e^−07^; ITS-SWARMS: p < 0.001; Fig. 4A,B). In contrast, the intraspecific variation in terms of ITS variants within V9-groups is higher in mountain ranges than in lowland areas (p < 0.001; Figure S4).

The fraction of OTUs exclusive to the respective altitudinal range is significantly higher in mountain ranges (V9-groups: p < 0.001; ITS-variants: p < 0.001; Fig. 4C,D) even though the absolute number of exclusive V9-groups and ITS-SWARMS is higher in the lowlands (654 V9-groups and 2,275 ITS-SWARMS are exclusive for mountain ranges, 11 675 V9-groups and 42 248 ITS-SWARMS are exclusive for lowland areas). An intermediate number of OTUs occurred independently of altitude (1 311 V9-groups and 1779 ITS-SWARMS occur in both regions).

### Geographic restriction and physicochemical differentiation of subpopulations as reflected by ITS-SWARMS

Approximately 70% of the ITS-SWARMS were only found in one lake (Table 1). Beyond the geographical patterns reflected by SSU sequences (V9-group level) a restricted geographical distribution of subpopulations (ITS-SWARMS associated with distinct V9-groups) could be confirmed (p < 0.001; Figure S5). The ratio of diversification within the ITS-SWARMS varied between the different taxonomic groups and nutritional strategies. In osmotrophic and saprophytic taxa, the number of ITS-SWARMS per V9-group was generally low (average number of 1.6–1.9) and the fraction of ITS-SWARMS with a geographical restriction was comparatively low, e.g. only 43% in Fungi and Chytridiomycetes and 47% in Oomycetes. In contrast, the predominantly phototrophic Stramenopiles, i.e. the Ochrophyta (Chrysophyceae, Bacillariophyceae, Dictyochophyceae, Eustigmatophyceae) generally had a higher number of ITS-SWARMS per V9-group (average number of 3.2–8.6) and a higher percentage of ITS-SWARMS with restricted geographical distributions (56–66%). Viridiplantae and Alveolata had an intermediate number of ITS-SWARMS per V9-group (average number of 2.2–2.6) and a low to intermediate geographical restriction of ITS-SWARMS (42% to 52% respectively). In all taxonomic groups some taxa included certain geographically restricted ITS-SWARMS (Figure S5^42^). Aside from indications for a geographical restriction of some subpopulations, the ITS-SWARMS also indicate an ecophysiological differentiation of clades below the resolution of V9-groups (Table 1). 3,242 V9-groups associated with the main taxon groups of interest were analyzed. The most significant differences of the examined V9-groups and their associated ITS-SWARM were found concerning altitudinal differences (77), followed by temperature and conductivity differences (76, respectively) and pH (44). Overviews on ecophysiological parameters of sampled lake are found in Figure S6.

## Discussion

Here, we reveal conspicuous patterns of protist endemism on a European scale, which differ between taxa. However, they do not necessarily correlate with patterns of protist taxon richness.

A surprisingly high number of V9-groups indicated a putatively restricted distribution (putatively endemics) (Fig. 1, Table 1) corresponding to the expectations derived from the moderate endemicity hypothesis^1,43,44^. Our results correspond to this hypothesis insofar as taxa (V9-groups) spanned the whole range from restricted to ubiquitous distribution.

The number of taxa with narrower distribution than expected by chance was surprisingly high and is not reflected by community studies, which report a significant but low effect of geography for structuring protist communities^14,18,45^. In contrast, many studies addressing the distribution of distinct species confirm a geographic restriction for numerous species (e.g.^4,11,43,46–48^) and different degrees of endemism are known for several taxa^12,49^. For instance, our data demonstrate that a large fraction of Chytridiomycetes have a restricted distribution (Figs. 1,3). This corresponds to a study from the Antarctic^50^. A high proportion of taxa with restricted distribution seems plausible for Chytridomycetes since many species are specific to a particular host or group of hosts (algae), further reducing the ability of wide dispersal^51^.

Our analyses show that distribution patterns of taxa with restricted distribution vary considerably both within and between taxonomic groups and do not follow a uniform regional pattern. Despite a high fraction of taxa with restricted distribution, patterns are therefore not or hardly visible at the community level as they are not consistent between taxa. This may explain the somewhat deviating view between taxon-specific investigations and community studies discussed above.

For protists the relations between taxon richness and centers of endemism have not, based on our knowledge, been investigated on a continental scale so far. Studies on animal and plant richness pattern imply that simple correlations are an oversimplification and that centers of endemism may be found both in areas with high and in areas with low or intermediate taxon richness^30,31^. However, for protists the distribution pattern of endemics and relations between the richness of taxa and of endemics may deviate from those known for macro-organisms. In particular, as patterns of taxon richness point to a lower importance of geographic barriers for protists, centers of endemism may also be less dependent on such barriers or even not exist at all (or simply reflect a fixed fraction of taxon richness).

Our data point to a low overlap between areas of increased taxon richness and increased endemism for protists. Indicating that centers of endemism (based on V9-groups) deviate from regions of high taxon richness. Rapid population growth and the combination of asexual and sexual reproduction could lead to a fast adaption to local environmental conditions^52^ and may therefore explain the observed missing overlap. For protist taxa with restricted distribution we revealed that distribution areas vary considerably, and boundaries of distribution areas do not necessarily coincide with geographic barriers such as mountain ranges (see additional information on https://doi.org/10.5281/zenodo.3674717). Thus, even though our data substantiate the hypothesis that geographic distance is relevant for dispersal, geographic barriers (which are important for structuring distribution ranges of macro-organisms^53^ ) seem to be of minor importance. However, it has to be considered that passive dispersal is limited by the dispersal capacity of the transport vector. It is beyond the scope of this study to analyze the differential adaptations between taxa with narrow and such having a wide distribution. Nevertheless, this aspect clearly is a promising objective for future research since our data indicate that dispersal limitations are important for protists even though the geographic barriers that restrict distribution ranges of macro-organisms may be of little relevance for protists.

High mountain ranges as azonal habitats harbor a deviating protistan diversity. Irrespective of their presumably low significance for protist dispersal, high mountain ranges are special habitats deviating in taxon richness and endemism from the surrounding lowlands^54^. Beyond a general decrease of taxon richness with altitude as predicted by species-area-relationships^55^, a common pattern for most protist taxa was a certain increase in the number of taxa exclusive to high mountain lakes (Fig. 4) and a certain cumulation of putatively endemic taxa within mountain ranges (Figs. 2, 3). Apart from the apparently low importance as barriers hindering dispersal, high mountain ranges provide azonal environmental conditions and thus can be expected to harbor taxa of restricted distribution and potentially endemic to the mountain range^33^. Further, we also observed an increase of taxa exclusive to high mountain lakes but not necessarily restricted to a distinct orogene. High mountain ecosystems are traditionally considered as extreme for life^56^. In high mountain lakes organisms are confronted with short growing seasons, low food nutrient availability or high incident solar radiation^57^. Harmful wavelengths of UV radiation can reach down to the lake bottom, in particular in clear water with low concentrations of humic substances and suspended particles^58,59^. The harsh environmental conditions of high mountain lakes require special adaptions to survive^14,60^ and may thus result in a high degree of taxa specializing on high mountain environment^61,62^. For instance, DNA repair mechanisms are important in adapting to high UV radiation levels and particularly for algae also photoprotection mechanisms^63^. The low nutrient availability and low water temperature further cause low densities of organisms and consequently low encounter rates which are require adaptations of foraging organisms to low food densities^14^.

Regions of high taxon richness (V9-richness) and centers of endemism differ between taxonomic groups. Apart from the azonal mountain habitats, we demonstrate that patterns of richness and endemism vary considerably between taxonomic groups. Patterns of two taxa, i.e. the Chrysophyceae and the Dinophyta may deserve special attention.

Taxon richness of Chrysophyceae was exceptionally high in Scandinavia, which corresponds to a pre-dominance of Chrysophyceae in many Scandinavian lakes^64^. Since many Scandinavian lakes are nutrient poor, slightly acidic and rich in humic substances the high taxon richness may reflect abiotic conditions favoring this taxonomic group^65–67^.

In contrast, the pattern of endemism was unexpected for Dinophyta. The number of putatively endemic taxa restricted to the area was particularly high near the Baltic Sea coastline while taxon richness in this area was only moderate. We can only speculate that due to the reduced salinity levels of the Baltic Sea^68^ the high degree of endemism in this area may result from a migration of taxa along environmental (salinity) gradients and subsequent speciation, while further dispersal might be limited by the niche width of individual taxa^69^. An increased phytoplankton diversity is known for the Baltic Bays and Bodden regions^70^ but it remains speculative why such a pronounced richness of putatively endemic taxa in this area was only found for Dinophyta. However, this region is strongly recommended for taxonomic follow-up studies as it likely holds numerous taxa waiting for discovery.

In contrast to these taxa with pronounced centers of endemism, diatoms had a remarkably low fraction of geographically restricted and endemic V9-groups, which may indicate either a generally wider distribution of diatom taxa or a general lack of taxonomic resolution in this group, i.e. that V9-groups reflect clusters of diatom species rather than individual species. This would support the observation that for diatoms the V9 region is well suited to explore genus-level diversity but has limited resolution at the species level^35^. The conspicuously low phylogenetic resolution within diatoms may be due to the fact that diatoms are a relatively young group, i.e. their diversification in the fossil record starting in the mid-mesozoic and their expansion paralleling the evolution and expansion of grasslands not before the mid-cenozoic^71,72^. Due to this (in geological terms) recent increase both in relative abundance and in diversity may be responsible for low phylogenetic resolution as the differentiation of gene sequences between species may be relatively recent.

Our results show that geographic restriction and physicochemical differentiation are already visible for subpopulations as reflected by ITS-SWARMS. We observed a generally high ITS-diversity. Subpopulations as reflected by ITS-SWARMS associated with given V9-groups provided meaningful information both with respect to geography and to abiotic adaptations. ITS sequence variation within V9 groups was particularly high in high mountain areas. Presumably, the loss of rare genotypes is lower and/or speciation rates are higher in mountain ranges as compared to lowland areas. This is not surprising since mutation rates are likely to be higher due to high UV radiation^73^ and population effects, e.g. the bottleneck effect, are presumably less important^74^ since generation times are likely to be prolonged due to the generally low temperatures and to nutrient limitations. Further, the pronounced environmental gradients and heterogeneous topographies may limit gene flow and colonization opportunities, thus leading to a stronger genetic differentiation^75,76^.

Interestingly, phototrophic and mixotrophic taxa exhibited a more pronounced geographical restriction of ITS-SWARMS than those of heterotrophic taxa. We assume that general differences in the growth and^77^ nutritional strategies are responsible for this pattern. Phototrophic taxa strongly depend on the surrounding physicochemical conditions^78^. Free living heterotrophic taxa are presumably less dependent on physicochemical conditions and thus less sensitive to changes. Niche specialization, requiring adaption and therefore genetic differentiation might therefore be infrequent in heterotrophs but frequent in phototrophs and mixotrophs. However, the differential geographic restriction of ITS-SWARMS between nutritional modes co-varied with the average number of ITS-SWARMS per V9 group. Differential dispersal can therefore not clearly be separated from differential diversity and sequence evolution and thus remains an issue for future research.

Contrary to our initial expectations, we could observe abiotic differentiation between subpopulations and it may, thus, be promising to include markers with higher phylogenetic resolution in future surveys of protist diversity. Independent on whether the ITS-SWARMS may represent species, which are not resolved by V9 variation or subspecies/subpopulations, we clearly demonstrate that ecological and geographical differentiation becomes evident on this level of resolution. Beyond the knowledge gain in basic reserach and the promises for protist population genetics our findings of a significant ecophysiological differentiation of subpopulations opens so far unexpected options for biomonitoring and the development of molecular and microbial bioindicators.

## Conclusion/summary

In summary, we demonstrated (i) that patterns of taxon richness and of endemism deviate for protists, (ii) that these patterns further deviate between different taxonomic groups, (iii) that high mountain ranges harbor a diverging protist diversity but presumably have only a low effect on protist dispersal as derived from distribution pattern in lowland areas, and (iv) that different taxonomic classes and phyla differ in microdiversity as reflected by ITS variants.

We demonstrate that geographic distance is relevant for protist dispersal but geographic barriers have only a low impact on structuring distribution pattern. These findings challenge the general validity of biogeographic patterns (mostly) derived from studies on macro-organisms. We thus propose that the proportion of the relative importance of geographic distance and of geographic barriers for structuring biodiversity on a continental scale systematically changes with size of the organism and biological traits (e.g., mode of nutrition). We further propose to incorporate this idea as dispersal disparity hypothesis and the respective scaling effects into future studies on differential distribution, dispersal and biogeography of organisms strongly differing in organismic size and in population size.

## Materials and methods

Eukaryotic amplicon sequences from 217 freshwater lakes across Europe (including Norway, Sweden, Germany, Poland, Romania, Austria, Italy, France, Spain, and Switzerland) were used in this study from the NCBI Bioproject PRJNA414052^18^. Sampling, DNA-isolation, sequencing and bioinformatic procession were conducted as described in detail in^18^. The sampled lakes reflect the predominant water body type in the different regions.

Briefly, samples were taken near the shore of the first 50 cm water column. Each lake was sampled once in august 2012 at one sampling point. For the DNA extraction and sequencing, the water was filtered onto 0.2 µm nucleopore filters, air dried and frozen in liquid nitrogen (Cryoshippers) and later stored at − 80 °C in the laboratory until further processing.Water temperature, pH and conductivity were determined directly in the field at the same sampling point and time by use of a Waterproof Tester “Combo” (Hanna Instruments, Vöhringen, Germany). Each measurement was performed 3 times and the mean value was calculated. The lakes were unequally distributed throughout Europe. The maximum distance (2,880,648 km) is between lake Nordmesna (Norway) and Embalse de Béznar (Spain); median distance is 781.045 km, mean distance 861.566 km with a standard deviation of 506.958 km.

The V9-ITS1 region of the 18S SSU and ITS region of the rDNA were amplified using a forward primer (5′-GTACACACCGCCCGTC-3′) and a combination of two reverse primers with different wobble positions (5′-GCTGCGCCCTTCATCGKTG-3′ (ITS2_Dino; 10%) and 5′-GCTGCGTTCTTCATCGWTR-3′ (ITS2_broad; 90%)) with an annealing temperature of 52 °C. Samples were equimolar pooled and commercially sequenced using an Illumina HiSeq 2,500 rapid run applying 2 × 300 bp reads with subsequent adapter trimming, quality trimming and demultiplexing (FASTERIS; Geneva, Switzerland). After quality filtering, assembly of reads and chimera removal^18^, sequences were filtered using the AmpliconDuo pipeline^79^. Sequencing results were dereplicated based on 100% identity including length variability (CD-HIT-EST algorithm;^80^). For each ITS-sequence, the most abundant assembled V9-sequence was kept as presumably correct sequence. Pairings of other V9-sequences with this specific ITS-sequence were excluded as likely erroneous sequence parings. Thus, each V9-sequence can be affiliated with multiple ITS-sequence pairings whereas each ITS-sequence after filtering was affiliated with only one distinct V9-sequence. Finally, reads were clustered based on identical V9-sequences (first 150 bp, R-Script “V9_Clust.R”;^81^), and taxonomically assigned based on the SSU fragment by searching the NCBI database using BLASTn (version 2.7.1). ITS sequences assigned to the same V9 sequence are denoted ITS variants of the respective V9. Reads assigned to Metazoans or higher plants were excluded from further analyses.

Samples with less than 15,069 reads after filtering, (corresponding to less than 15% of the median number of reads per lake) were excluded from further analysis. ITS-variants with a read abundance of less than 0.001% of total reads per lake (corresponding roughly to 10 reads per lake) were also excluded for the corresponding lake. Remaining ITS-variants belonging to the identical V9-sequences were independently clustered using SWARM (= ITS-SWARM; version 2.2.2;^82^). All subsequent statistical analyses were based on presence-absence data of V9-groups or ITS-SWARMS using the software R (version 3.6.0;^83^).

### Restrictive geographical distribution of V9-groups

All V9-groups, which occurred in at least two lakes, were used for identifying areas harboring high numbers of taxa with potentially restricted distribution. For each of these V9-groups the average distances between lakes in which the respective V9-group occurred was calculated and tested using a paired t-test against the average distance between an equal number of randomly selected lakes (based on 1,000 random drawings per V9-group); a spatial distance matrix was calculated based on the GPS data using distm() and the “distHaversine” option (geosphere; version 1.5–10;^84^).

In the context of this study we considered a V9-group (or ITS variant) as being geographically restricted when the mean distance between the lakes harboring this V9-group (or ITS variant) was smaller than would have been expected for random distribution. Further, in the context of this study we considered a V9-group as putatively endemic when two criteria were met: (i) the distance of lakes that contain a given V9-group was smaller than that of randomly chosen lakes in at least 90% of the simulations (based on 1,000 simulations as outlined above) and (ii) all lakes that contained the given V9-group were found within a maximum (rectangular) area of 1,000,000 km .

*Regions of high richness and regions harboring a high number of V9-groups with restricted distribution* were analyzed based on a geographic grid with a grid size of 2.5° longitude × 2.5° latitude. The number of lakes per grid are displayed in Figure S7. Regional diversity was independently analyzed for all taxonomic groups (classes or phyla) comprising at least 50 V9-groups (Apicomplexa, Bacillariophyceae, Cercozoa, Viridiplantae, Chrysophyceae, Chytridiomycetes, Ciliophora, Dictyochophyceae, Dinophyceae, Eustigmatophyceae, Fungi, Oomycetes). In order to account for different numbers of lakes per grid, we calculated an interpolated number of OTUs per lake for each taxonomic group and each grid (N_interpolated_), i.e. we applied non-linear regression (N_interpolated_ = a * log(N + 1) + b with N: number of lakes) to rarefaction curves for each grid separately. Prior tests demonstrated that the chosen regression formula reflected best the relation between OTU number and number of lakes (data not shown). Based on this regression we calculated for each grid and each taxonomic group the expected number of OTUs for one lake in the respective grid, for grids containing a single lake only the original number of OTUs was used.

Differences between low-altitude and high mountain lakes were assessed by comparing lakes at high elevations (above 1,500 m altitude) and lakes below 1,500 m altitude (Figure S8 shows the largest mountain ranges in the study area). Total numbers of V9-groups and ITS-SWARMS per lake, as well as the number of ITS- SWARMS per V9-group were compared using the Wilcoxon-test. Since the total number of low-altitude lakes was much higher than that of high mountain lakes (n = 27), the exclusiveness of V9-groups and ITS-SWARMS in low-altitude and mountain lakes was compared using equivalent numbers of low-altitude lakes (n = 27), i.e. subsets of randomly chosen low-altitude lakes (based on 100 randomly permutated drawings; the diversity of both, lowland and high mountain lakes was tested against the remaining lowland lakes (n = 127)).

*Geographic restriction and ecophysiological adaptation of subpopulations based on ITS-SWARMS assigned to distinct V9-groups *was analyzed by simulations and paired t-tests (as outlined above for V9-groups). For geographic restriction, the average distances between lakes were compared for all ITS-SWARMS, which occurred in at least two different lakes, and tested against average distances between equal numbers of random lake combinations (1,000 random drawing per ITS-SWARM; for these analyses all lakes were considered as reference in which the respective V9-group occurred). Analyses concerning the differential distribution of ITS-SWARMS with respect to physicochemical factors were further restricted to V9-groups with at least two associated ITS-SWARMS. Differences in environmental parameters (pH, conductivity, temperature and altitude) between lakes affiliated with a distinct ITS-SWARMS of one V9-group were tested using Kruskal-Wallice tests^85^ followed by Dunns-Test^86^.

## Supplementary information

Supplementary information.

## Data Availability

Eukaryotic amplicon sequences from 217 natural freshwater lakes across Europe were used in this study from the NCBI Bioproject PRJNA414052^18^. Additional material (distribution maps of V9 groups) can be found under https://doi.org/10.5281/zenodo.3674717.

## References

[1] Segawa T (2018). Bipolar dispersal of red-snow algae. Nat. Commun..

[2] Tedersoo L (2014). Fungal biogeography. Global diversity and geography of soil fungi. Science (New York, N.Y.).

[3] Dunthorn M, Stoeck T, Wolf K, Breiner H-W, Foissner W (2012). Diversity and endemism of ciliates inhabiting Neotropical phytotelmata. Syst. Biodivers..

[4] Siver PA, Lott AM (2012). Biogeographic patterns in scaled chrysophytes from the east coast of North. America.

[5] Gaston KJ (2000). Global patterns in biodiversity. Nature.

[6] Bass D, Boenigk J, Fontaneto D, Fontaneto D (2011). Biogeography of Microscopic Organisms.

[7] Caron DA (2009). Past President's address: protistan biogeography: why all the fuss?. J. Eukaryot. Microbiol..

[8] Foissner, W. Biogeography and dispersal of micro-organisms: a review emphasizing protists (2006).

[9] Coesel PFM, Krienitz L (2008). Diversity and geographic distribution of desmids and other coccoid green algae. Biodivers. Conserv..

[10] Darling KF, Wade CM (2008). The genetic diversity of planktic foraminifera and the global distribution of ribosomal RNA genotypes. Mar. Micropaleontol..

[11] Vanormelingen P, Verleyen E, Vyverman W (2008). The diversity and distribution of diatoms: from cosmopolitanism to narrow endemism. Biodivers. Conserv..

[12] Stoeck T, Bruemmer F, Foissner W (2007). Evidence for local ciliate endemism in an alpine anoxic lake. Microbiol. Ecol..

[13] Fernández LD, Hernández CE, Schiaffino MR, Izaguirre I, Lara E (2017). Geographical distance and local environmental conditions drive the genetic population structure of a freshwater microalga (Bathycoccaceae; Chlorophyta) in Patagonian lakes. FEMS Microbiol. Ecol..

[14] Filker S, Sommaruga R, Vila I, Stoeck T (2016). Microbial eukaryote plankton communities of high-mountain lakes from three continents exhibit strong biogeographic patterns. Mol. Ecol..

[15] de Vargas C (2015). Eukaryotic plankton diversity in the sunlit ocean. Science.

[16] Ibarbalz FM (2019). Global trends in marine plankton diversity across kingdoms of life. Cell.

[17] Bock C, Salcher M, Jensen M, Pandey RV, Boenigk J (2018). Synchrony of eukaryotic and prokaryotic planktonic communities in three seasonally sampled Austrian lakes. Front. Microbiol..

[18] Boenigk J (2018). Geographic distance and mountain ranges structure freshwater protist communities on a European scale. Metabarcoding and Metagenomics.

[19] He F (2020). Elevation, aspect, and local environment jointly determine diatom and macroinvertebrate diversity in the Cangshan Mountain, Southwest China. Ecol. Indic..

[20] Shen C (2014). Contrasting elevational diversity patterns between eukaryotic soil microbes and plants. Ecology.

[21] Bryant JA (2008). Colloquium paper: microbes on mountainsides: contrasting elevational patterns of bacterial and plant diversity. Proc. Natl. Acad. Sci. USA.

[22] McCain CM (2007). Could temperature and water availability drive elevational species richness patterns? A global case study for bats. Global Ecol. Biogeogr..

[23] Desmond A (1983). Janet Browne, The secular ark. Studies in the history of biogeography, New Haven, Conn., and London, Yale University Press, 1983, 8vo, pp. x, 273, illus., £21.00. Med. Hist..

[24] Nemcová Y, Kreidlová J, Kosová A, Neustupa J (2012). Lakes and pools of Aquitaine region (France)—a biodiversity hotspot of Synurales in Europe. Nova Hedw.

[25] van de Vijver B, Gremmen NJM, Beyens L (2005). The genus Stauroneis (Bacillariophyceae) in the Antarctic region. J. Biogeogr..

[26] Martiny JBH (2006). Microbial biogeography: putting microorganisms on the map. Nat. Rev. Microbiol..

[27] Lepère C (2013). Geographic distance and ecosystem size determine the distribution of smallest protists in lacustrine ecosystems. FEMS Microbiol. Ecol..

[28] Green JL (2004). Spatial scaling of microbial eukaryote diversity. Nature.

[29] Lara E, Roussel-Delif L, Fournier B, Wilkinson DM, Mitchell EAD (2016). Soil microorganisms behave like macroscopic organisms: patterns in the global distribution of soil euglyphid testate amoebae. J. Biogeogr..

[30] Kier G (2009). A global assessment of endemism and species richness across island and mainland regions. Proc. Natl. Acad. Sci. USA.

[31] Orme CDL (2005). Global hotspots of species richness are not congruent with endemism or threat. Nature.

[32] Schmitt T (2009). Biogeographical and evolutionary importance of the European high mountain systems. Front. Zool..

[33] Vimercati L, Darcy JL, Schmidt SK (2019). The disappearing periglacial ecosystem atop Mt. Kilimanjaro supports both cosmopolitan and endemic microbial communities. Sci. Rep..

[34] McCain CM (2005). Elevational gradients in diversity of small mammals. Ecology.

[35] Malviya S (2016). Insights into global diatom distribution and diversity in the world's ocean. Proc. Natl. Acad. Sci. USA.

[36] Khomich M, Kauserud H, Logares R, Rasconi S, Andersen T (2017). Planktonic protistan communities in lakes along a large-scale environmental gradient. FEMS Microbiol. Ecol..

[37] Škaloud P (2019). Speciation in protists: Spatial and ecological divergence processes cause rapid species diversification in a freshwater chrysophyte. Mol. Ecol..

[38] Godhe A, McQuoid MR, Karunasagar I, Karunasagar I, Rehnstam-Holm A-S (2006). Comparison of three common molecular tools for distinguishing among geographically separated clones of the diatom Skeletonema marinoi sarno et zingone (Bacillariophyceae). J. Phycol..

[39] Jobst J, King K, Hemleben V (1998). Molecular evolution of the internal transcribed spacers (ITS1 and ITS2) and phylogenetic relationships among species of the family cucurbitaceae. Mol. Phylogenet. Evol..

[40] Lobo-Hajdu G (2004). Intragenomic, intra- and interspecific variation in the rDNA its of Porifera revealed by PCR-singlestrand conformation polymorphism (PCR-SSCP). Bollettino dei Musei e degli Istituti Biologici.

[41] Needham DM, Sachdeva R, Fuhrman JA (2017). Ecological dynamics and co-occurrence among marine phytoplankton, bacteria and myoviruses shows microdiversity matters. ISME J..

[42] Derot J (2020). Response of phytoplankton traits to environmental variables in French lakes: new perspectives for bioindication. Ecol. Indic..

[43] Boo SM (2010). Complex phylogeographic patterns in the freshwater alga Synura provide new insights into ubiquity vs. endemism in microbial eukaryotes. Mol. Ecol..

[44] Foissner W, Hawksworth DL (2009). Protist Diversity and Geographical Distribution.

[45] Schiaffino MR (2016). Microbial eukaryote communities exhibit robust biogeographical patterns along a gradient of Patagonian and Antarctic lakes. Environ. Microbiol..

[46] Boenigk J (2006). Evidence for geographic isolation and signs of endemism within a protistan morphospecies. Appl. Environ. Microbiol..

[47] Foissner W, Chao A, Katz LA (2008). Diversity and geographic distribution of ciliates (Protista: Ciliophora). Biodivers. Conserv..

[48] Payo DA (2013). Extensive cryptic species diversity and fine-scale endemism in the marine red alga Portieria in the Philippines. Proc. R. Soc. B.

[49] Siver PA, Skogstad A, Nemcová Y (2019). Endemism, palaeoendemism and migration: the case for the ‘European endemic’, Mallomonas intermedia. Eur. J. Phycol..

[50] Cox F, Newsham KK, Robinson CH (2019). Endemic and cosmopolitan fungal taxa exhibit differential abundances in total and active communities of Antarctic soils. Environ. Microbiol..

[51] Ibelings BW (2004). Host parasite interactions between freshwater phytoplankton and chytrid fungi (Chytridiomycota). J. Phycol..

[52] Logares R (2009). Infrequent marine-freshwater transitions in the microbial world. Trends Microbiol..

[53] Hewitt GM (2004). The structure of biodiversity—insights from molecular phylogeography. Front. Zool..

[54] Vetaas OR, Grytnes J-A (2002). Distribution of vascular plant species richness and endemic richness along the Himalayan elevation gradient in Nepal. Global Ecol. Biogeogr..

[55] Nogués-Bravo D, Araújo MB, Romdal T, Rahbek C (2008). Scale effects and human impact on the elevational species richness gradients. Nature.

[56] Catalán J, Camarero L, Felip M, Pla SC, Ventura M, Buchaca T, Bartumeus F, Mendoza G, Miró A, Casamayor EO, Medina-Sánchez JM, Bacardit M, Altuna M, Bartrons M, Quijano DD (2006). High mountain lakes: extreme habitats and witnesses of environmental changes. Limnetica.

[57] Sommaruga R (2001). The role of solar UV radiation in the ecology of alpine lakes. J. Photochem. Photobiol. B Biol..

[58] Morris DP (1995). The attenuation of solar UV radiation in lakes and the role of dissolved organic carbon. Limnol. Oceanogr..

[59] Sommaruga R, Augustin G (2006). Seasonality in UV transparency of an alpine lake is associated to changes in phytoplankton biomass. Aquat. Sci..

[60] Catalan J (2006). High mountain lakes: extreme habitats and witnesses of environmental changes. Limnética.

[61] Ortiz-Álvarez R, Triadó-Margarit X, Camarero L, Casamayor EO, Catalan J (2018). High planktonic diversity in mountain lakes contains similar contributions of autotrophic, heterotrophic and parasitic eukaryotic life forms. Sci. Rep..

[62] Kammerlander B (2015). High diversity of protistan plankton communities in remote high mountain lakes in the European Alps and the Himalayan mountains. FEMS Microbiol. Ecol..

[63] Tartarotti B (2014). UV-induced DNA damage in Cyclops abyssorum tatricus populations from clear and turbid alpine lakes. J. Plankton Res..

[64] Brettum P, Halvorsen G (2004). The phytoplankton of Lake Atnsjøen, Norway—a long-term investigation. Hydrobiologia.

[65] Karlsson J (2009). Light limitation of nutrient-poor lake ecosystems. Nature.

[66] Bergström A-K, Karlsson D, Karlsson J, Vrede T (2015). N-limited consumer growth and low nutrient regeneration N: P ratios in lakes with low N deposition. Ecosphere.

[67] Kritzberg ES (2020). Browning of freshwaters: consequences to ecosystem services, underlying drivers, and potential mitigation measures. Ambio.

[68] Gustafsson BG, Westman P (2002). On the causes for salinity variations in the Baltic Sea during the last 8500 years. Paleoceanography.

[69] Filker S, Kühner S, Heckwolf M, Dierking J, Stoeck T (2019). A fundamental difference between macrobiota and microbial eukaryotes: protistan plankton has a species maximum in the freshwater-marine transition zone of the Baltic Sea. Environ. Microbiol..

[70] Schiewer U, Schiewer U (2008). Ecology of Baltic Coastal Waters.

[71] Falkowski PG (2004). The evolution of modern eukaryotic phytoplankton. Science (New York, N.Y.).

[72] Cermeño P, Falkowski PG, Romero OE, Schaller MF, Vallina SM (2015). Continental erosion and the Cenozoic rise of marine diatoms. Proc. Natl. Acad. Sci. USA.

[73] Rothschild LJ (1999). The influence of UV radiation on protistan evolution. J. Eukaryot. Microbiol..

[74] Rose JM, Caron DA (2007). Does low temperature constrain the growth rates of heterotrophic protists? Evidence and implications for algal blooms in cold waters. Limnol. Oceanogr..

[75] Ægisdóttir HH, Kuss P, Stöcklin J (2009). Isolated populations of a rare alpine plant show high genetic diversity and considerable population differentiation. Ann. Bot..

[76] Cain ML, Milligan BG, Strand AE (2000). Long-distance seed dispersal in plant populations. Am. J. Bot..

[77] Nemcová Y, Pichrtova M (2012). Shape dynamics of silica scales (Chrysophyceae, Stramenopiles) associated with pH. Fottea.

[78] Leadbeater BSC, Green JC (2000). Flagellates: Unity, Diversity and Evolution. Chapter 12: Functional Diversity of Heterotrophic Flagellates in Aquatic Ecosystems.

[79] Lange A (2015). AmpliconDuo: a split-sample filtering protocol for high-throughput amplicon sequencing of microbial communities. PLoS ONE.

[80] Fu L, Niu B, Zhu Z, Wu S, Li W (2012). CD-HIT: accelerated for clustering the next-generation sequencing data. Bioinformatics.

[81] Jensen, M. V9_Clust.R. R-Scrift for modifying DNA-sequence-abundance-tables: clustering of related sequences (e.g. SSU-ITS1) according to 100% identical subsequences. https://github.com/manfred-uni-essen/V9-cluster (2017).

[82] Mahé F, Rognes T, Quince C, de Vargas C, Dunthorn M (2015). Swarm v2: highly-scalable and high-resolution amplicon clustering. PeerJ.

[83] R Core Team. R: A language and environment for statistical computing (2019).

[84] Hijmans, R. J. *Spherical Trigonometry [R package geosphere version 1.5–10]* (2019).

[85] Kruskal WH, Wallis WA (1952). Use of ranks in one-criterion variance analysis. J. Am. Stat. Assoc..

[86] Dunn OJ (1961). Multiple comparisons among means. J. Am. Stat. Assoc..

