# Supplementary material for: Centers of endemism of freshwater protists deviate from pattern of taxon richness on a continental scale
Source: Sci Rep. 2020 Sep 2;10:14431. doi: 10.1038/s41598-020-71332-z (PMC7468153; doi:10.1038/s41598-020-71332-z)
Supplement: Supplementary file 1 — Supplementary information. [file 41598_2020_71332_MOESM1_ESM.pdf]

Supplemental material:

Centers of endemism of freshwater protists deviate from pattern of taxon richness on a continental scale

Jana L. Olefeld[1], Christina Bock[1], Manfred Jensen[1], Janina C. Vogt[2], Guido Sieber[1], Dirk Albach[2], Jens Boenigk[1]

[1] Biodiversity, University of Duisburg-Essen, Universitätsstr. 5, 45141 Essen, Germany

[2] Institute for Biology and Environmental Science (IBU), Plants Biodiversity and Evolution, Carl von Ossietzky University, Carl-von-Ossietzky-Str. 9-11, 26129 Oldenburg, Germany

Corresponding author: Jens Boenigk, Biodiversity, University of Duisburg-Essen, Universitätsstr. 5, 45141 Essen, Germany

Email: jens.boenigk@uni-due.de

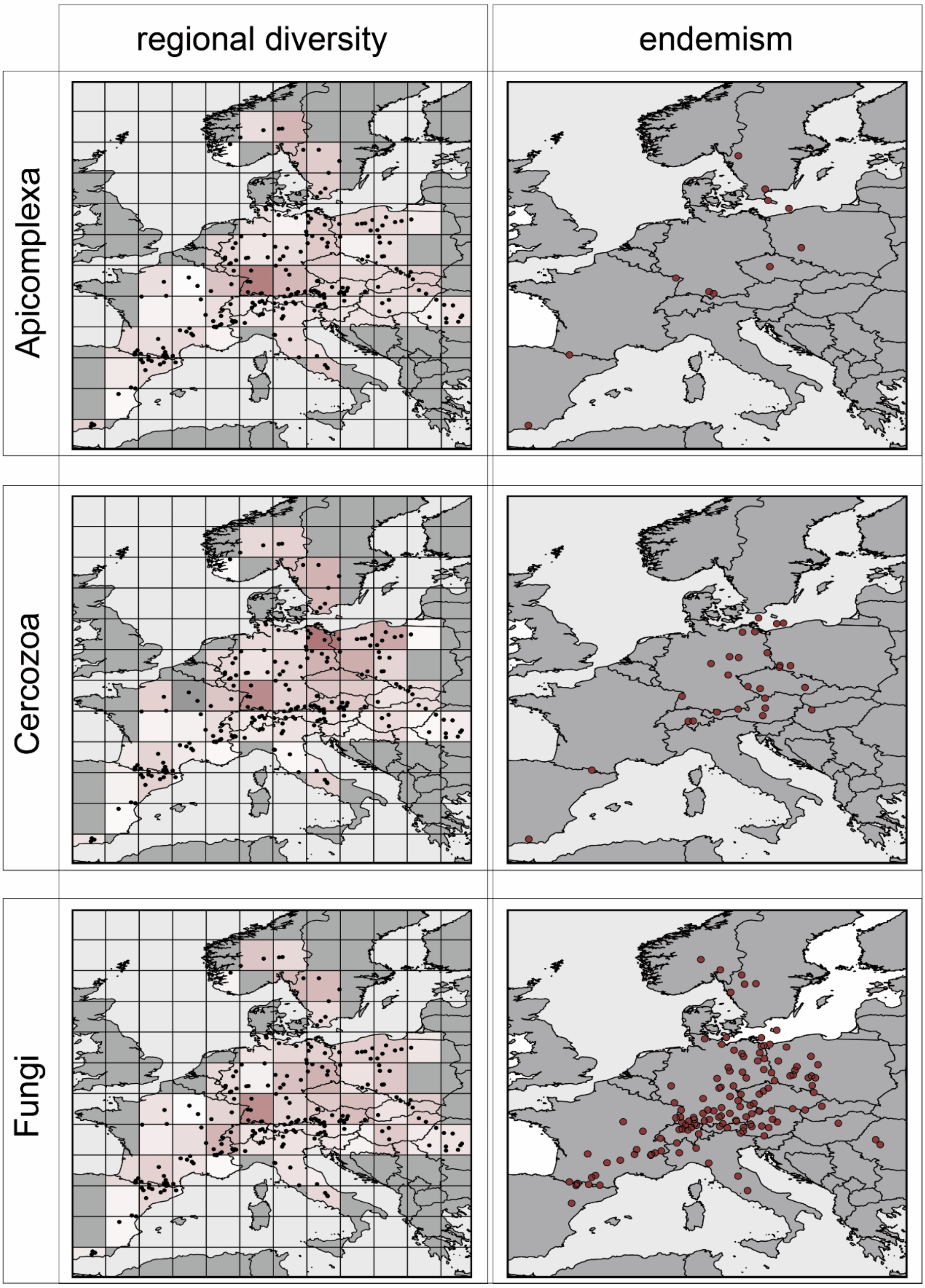

Figure S1: Regional diversity of Apicomplexa, Cercozoa and Fungi. Left: Regional richness of the respective taxon where color shading indicates low (white) to high (red) regional richness. Maximal shading corresponds to 16.2 interpolated V9-groups per lake for Apicomplexa, to 17.3 interpolated V9-groups per lake for Cercozoa and to 14.2 interpolated V9-groups per lake for Fungi. Right: centers of distribution areas for putatively endemic taxa within the respective group. Please note that the centers of the distribution area are geometric centers and do not coincide with a location of a distinct lake.

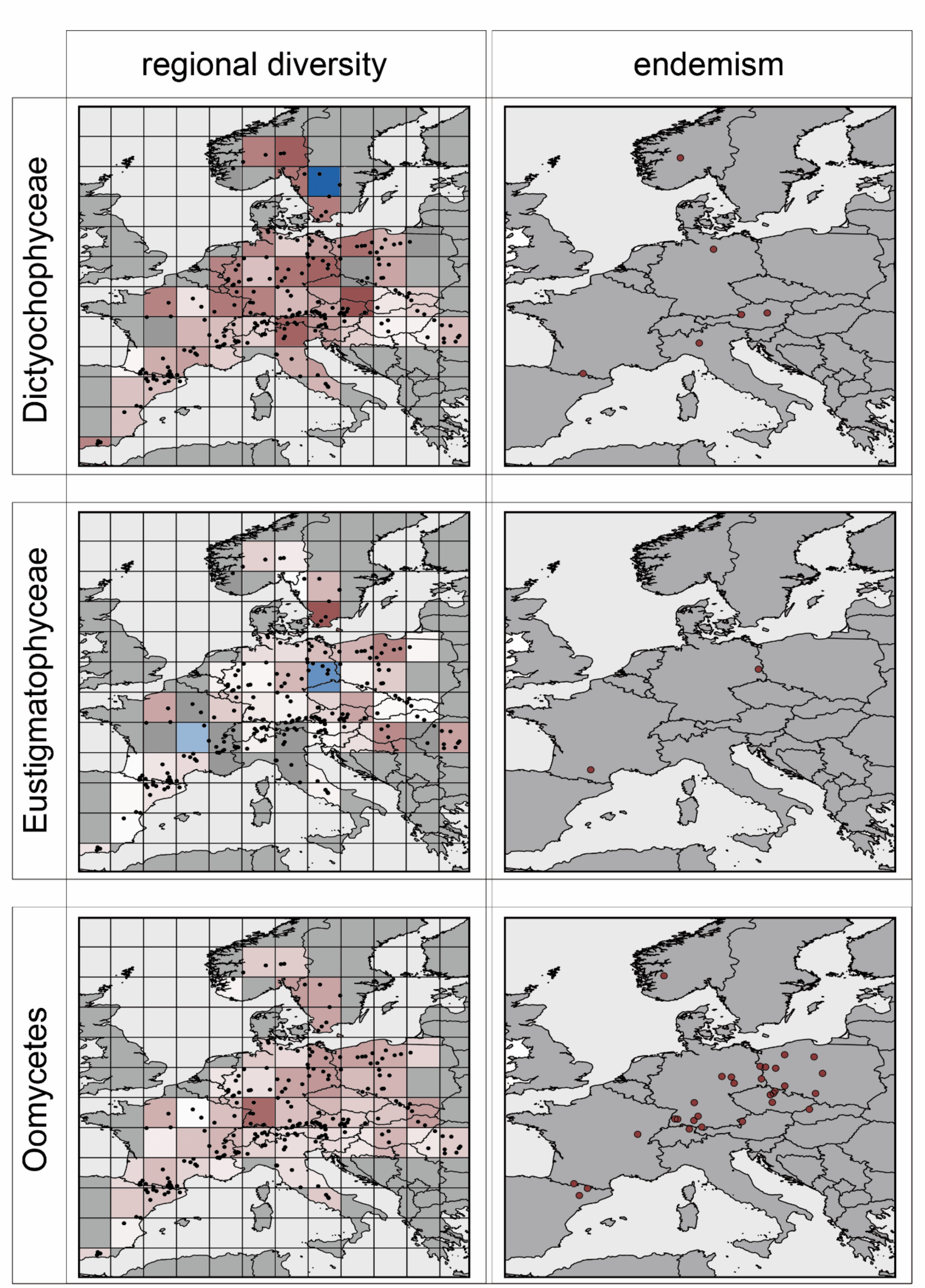

Figure S2: Regional diversity of Dictyochophytes, Eustigmatophytes and Oomycetes. Left: Regional richness of the respective taxon where color shading indicates low (white) to high (red) regional richness (blue shading indicates an exceptionally high richness). Maximal shading corresponds to 41.8 (blue) for Dictyochophyceae, to 34.5 (blue) for Eustigmatophyceae and 18.5 for Oomycetes. Right: centers of distribution areas for putatively endemic taxa within the respective group. Please note that the centers of the distribution area are geometric centers and do not coincide with a location of a distinct lake.

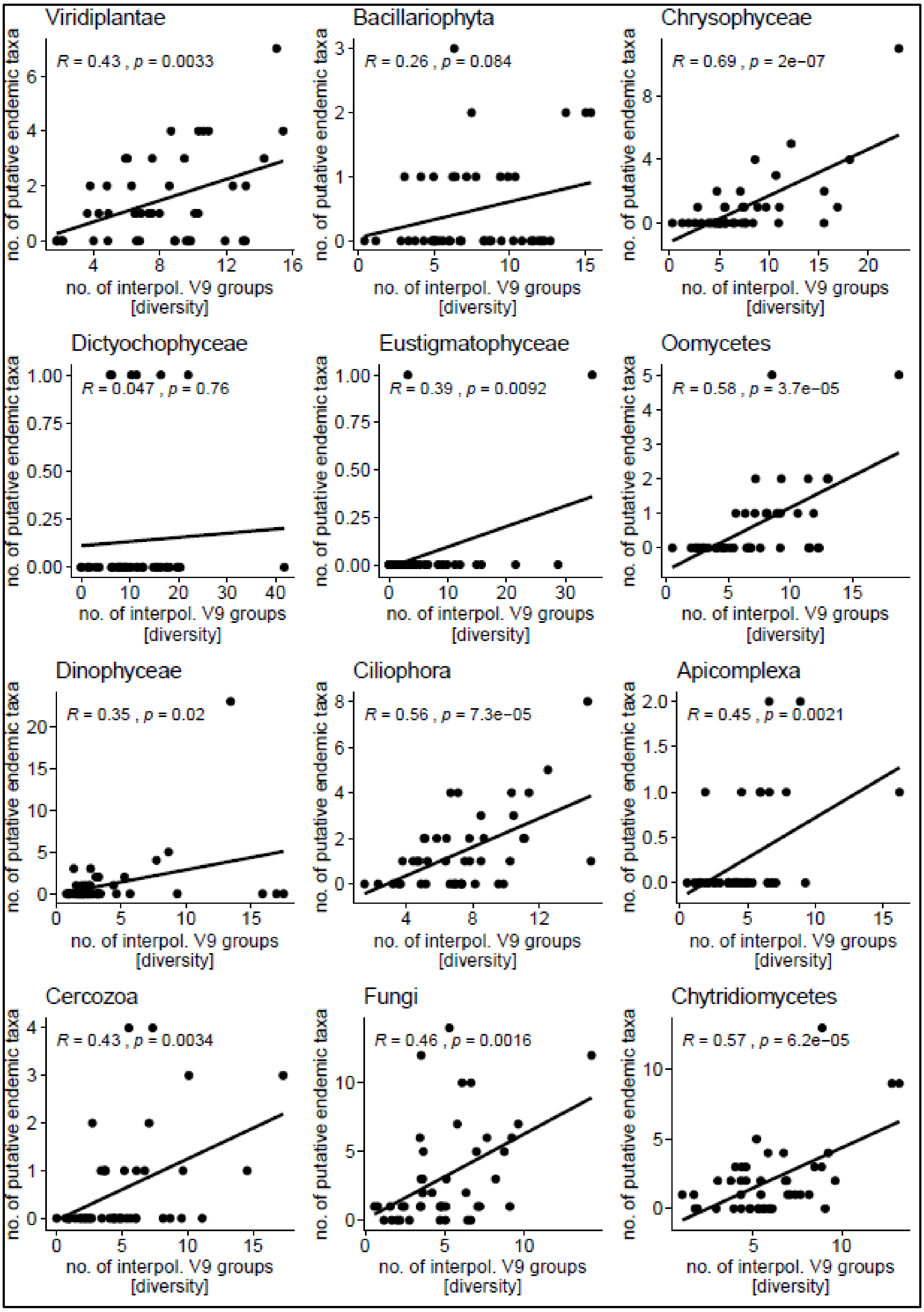

Figure S3

Correlation between diversity of the interpolated V9 groups per grid and putatively endemic centers within different taxonomic groups (visually displayed in Figures 2, 3, S1, S2). Pearson's correlation coefficient (R) was calculated using ggpubr' (vs 0.2.3) and tested for significance (*p*).

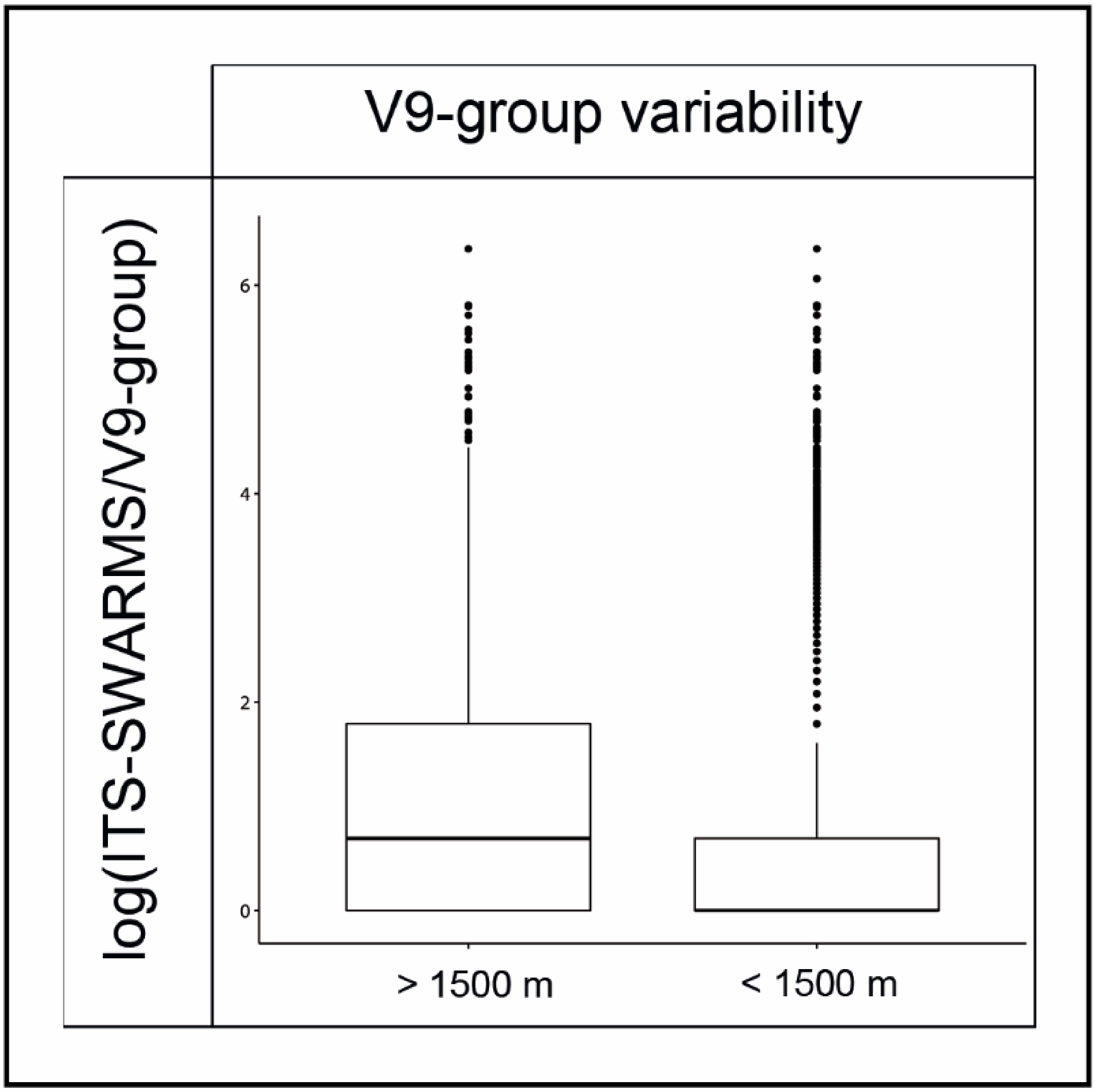

Figure S4: Microdiversity of V9-groups, i.e. the number of ITS-SWARMS affiliated with a distinct V9 group, in lakes above and below 1500m.

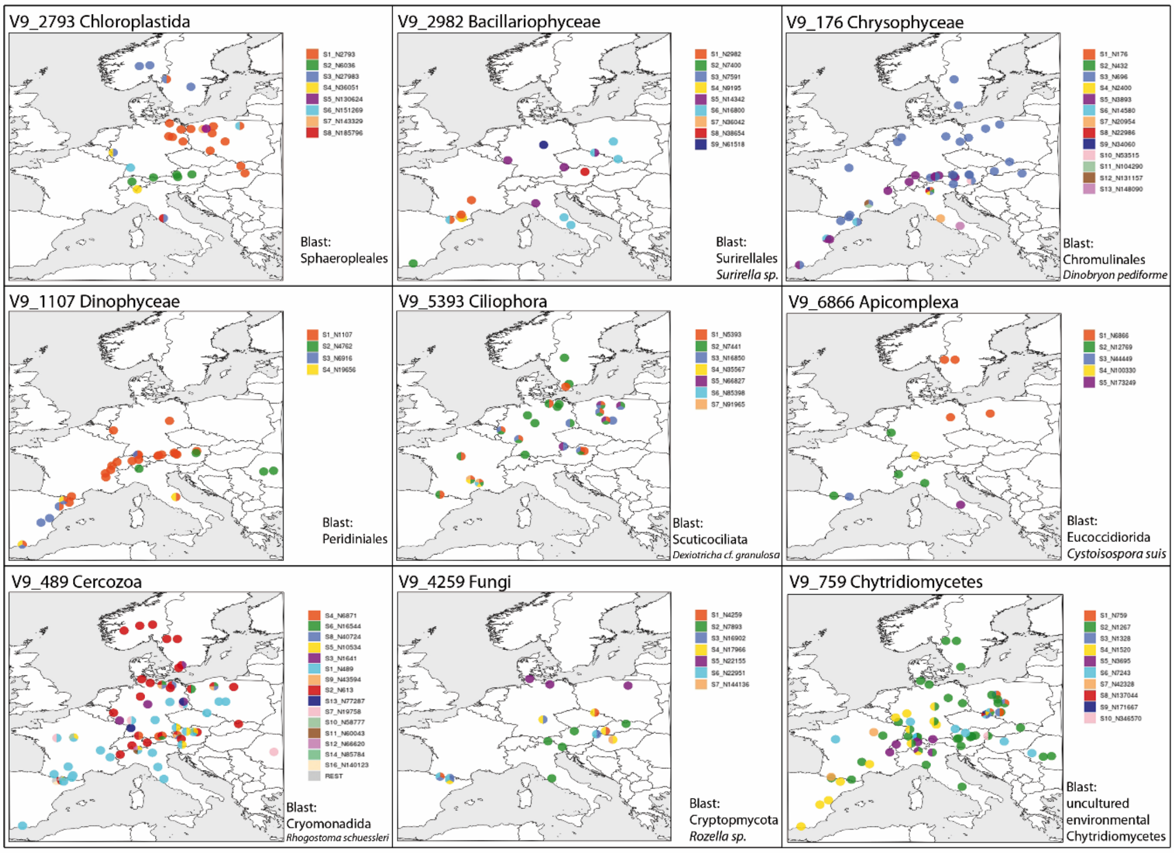

Figure S5: Examples for V9 groups affiliated with different phyla which show a geographically differential distribution of associated ITS_SWARMS.

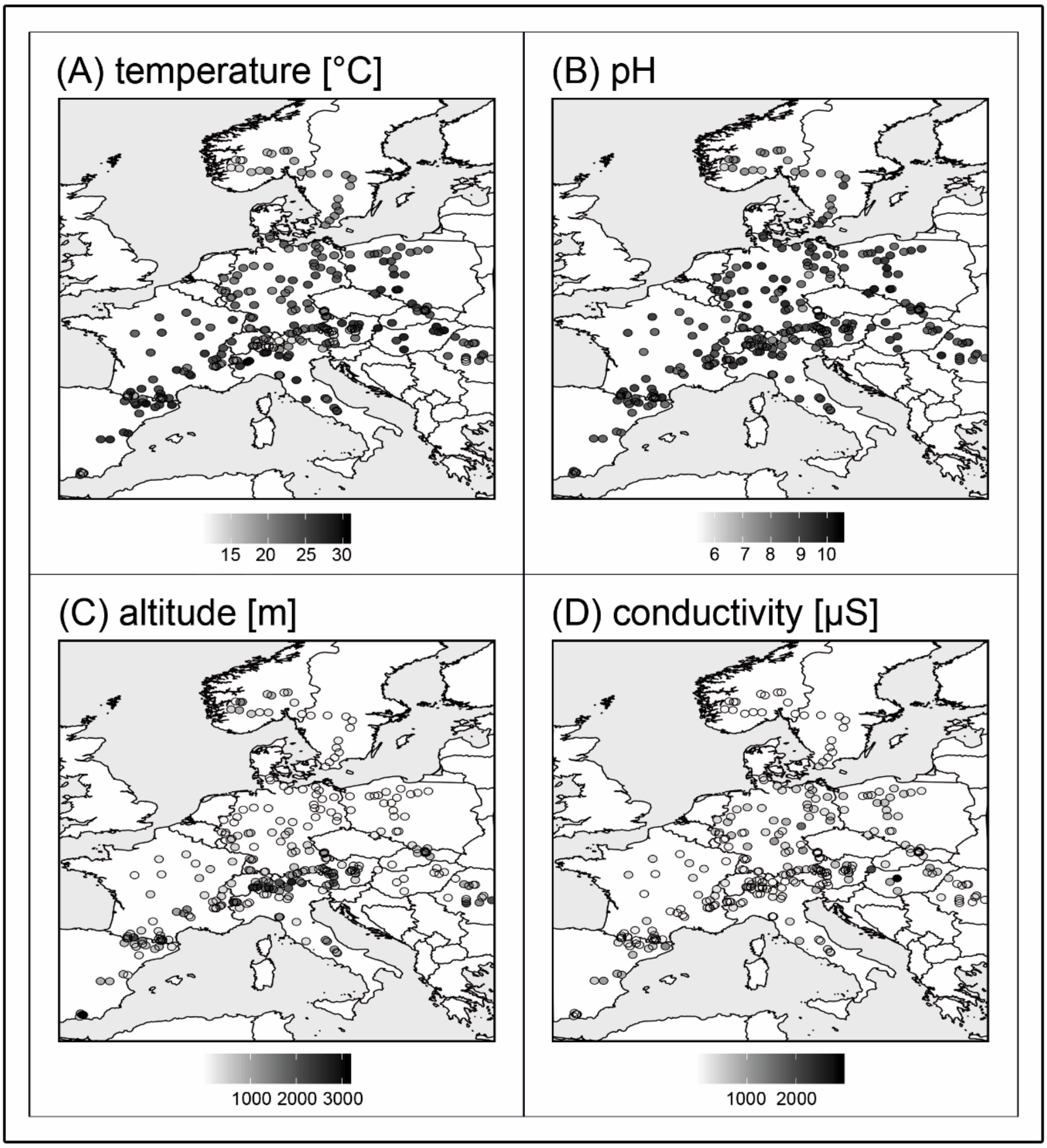

Figure S6: Temperature, pH, altitude and conductivity for the sampling sites.

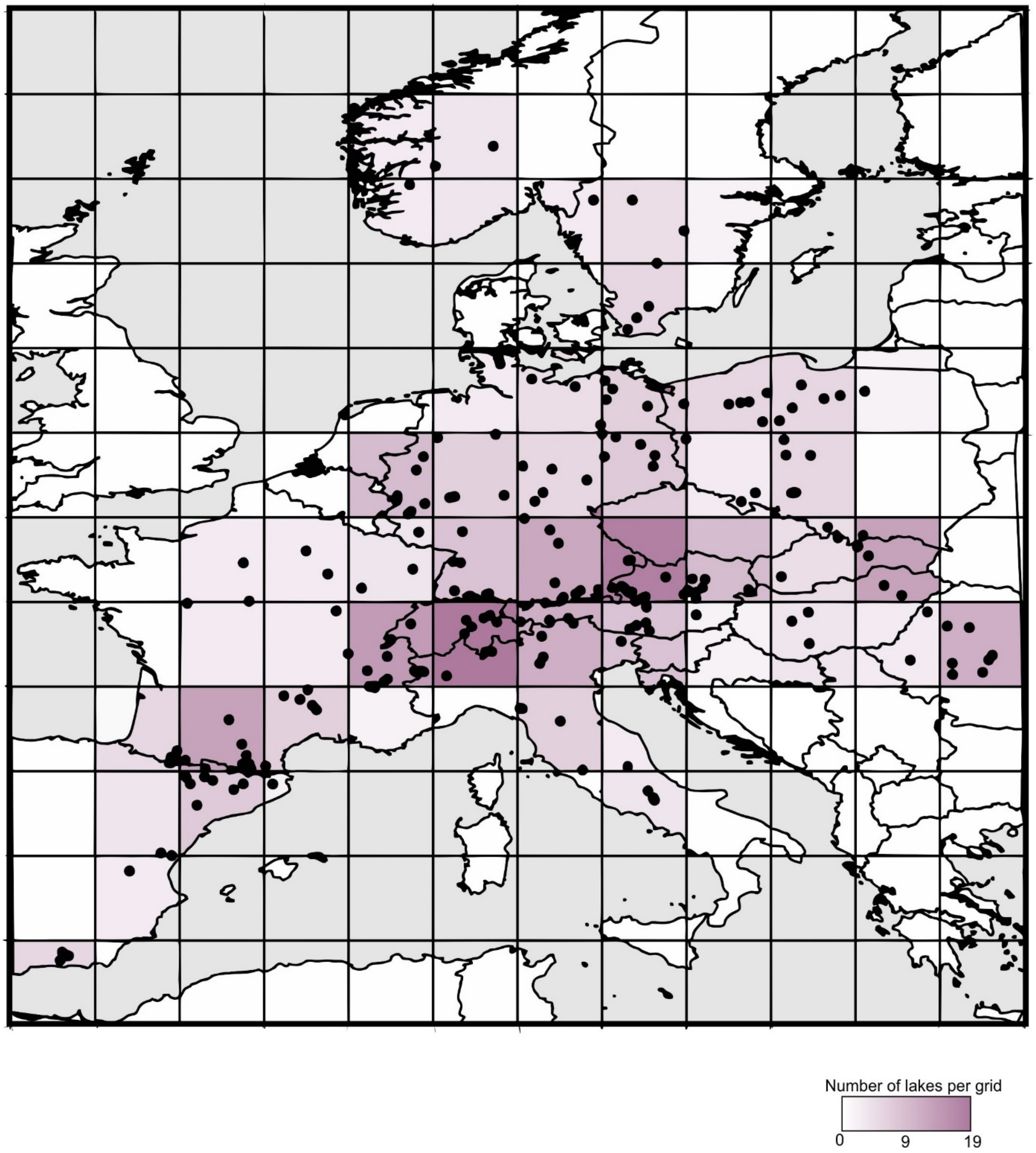

Figure S7:

Regional distribution of the sampled lakes. Shading corresponds to number of lakes per grid.

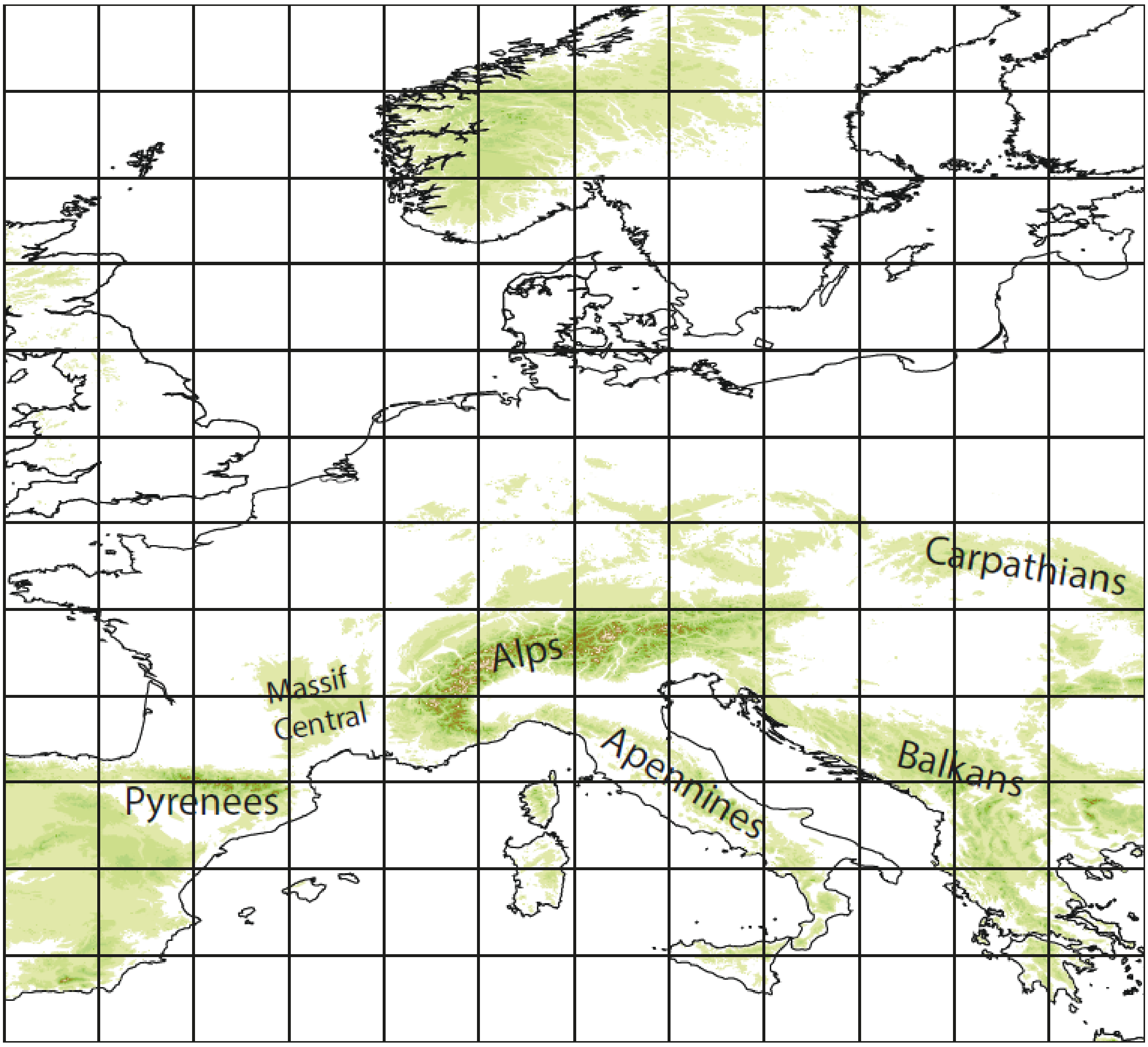

Figure S8

Geographical elevations within the sampling area.
